# The importance of daily removal of the denture biofilm for oral and systemic diseases prevention

**DOI:** 10.1590/1678-77572015ed006

**Published:** 2015

**Authors:** Karin Hermana Neppelenbroek

Dear Readers,

Inadequate cleaning of removable dentures promotes the accumulation and adhesion of biofilm, which is one of the main causes of failure of these prostheses. Complete denture biofilm is defined as a dense layer of complex microbial communities embedded in a polymeric matrix, and it is known to contain more than 1011 microorganisms by gram in dry weight^13^. The composition of the denture biofilm microbial community is similar to that of dental biofilm with the exception of an increase in *Candida* spp^13^.

It has been widely reported that denture biofilm acts as a reservoir for opportunistic microorganisms that can cause local infections, especially *Candida*-related denture stomatitis, or even systemic diseases^1^ ^,^ ^3^ ^,^ ^8^ ^,^ ^15^. Denture stomatitis is the most common form of oral candidal infection found on the palate of denture wearers (11 to 70%)^3^ ^,^ ^18^. About 93% of individuals with clinical signs of denture stomatitis present fungal infection, being *Candida albicans* the most prevalent species, identified in 50 to 98% of all cases^2^ ^,^ ^18^. *Candida* spp. interact with other microorganisms in the oral cavity, particularly *Streptococcus* spp. and *Staphylococcus* spp., thus resulting in a complex and mixed biofilm formation with organized structure that is difficult to remove^2^ ^,^ ^18^. Moreover, bacteria in acrylic biofilms resulted in higher virulence and pathogenicity of *Candida* biofilms^4^. Apart from local infections such as denture stomatitis, recent studies have recognized a relationship between denture biofilm and systemic diseases, mainly in elderly individuals^8^ ^,^ ^15^. Kashiwabara, et al.^8^ (2007) found high levels of *Candida* spp. and *Staphylococcus* spp. (including methicillin-resistant *Staphylococcusaureus*) from palatal tissue surface of maxillary dentures of geriatric inpatients and maxillary defective patients, respectively. It is know that respiratory pathogens preferentially colonize teeth or dentures, rather than soft tissue, being the pneumonia the leading cause of death attributable to infection in patients aged 65 years and older^5^. According to O’Donnell, et al.^15^ (2015), given a denture’s close proximity to the respiratory tract, denture wearers are potentially at an increased risk of aspirating opportunistic pathogens from the denture into their lungs. These authors showed a high prevalence of putative respiratory pathogens on the dentures of ambulatory adults, a finding that could explain the source of infection in some cases of aspiration pneumonia^16^. Besides aspiration pneumonia, oral pathogens have been implicated in bacterial endocarditis, gastrointestinal infection and chronic obstructive pulmonary disease, among others, and dentures offer a reservoir for microorganisms associated with these infections^5^ ^,^ ^8^. Therefore, it is crucial that careful daily removal of the biofilm present in the oral cavity and on complete dentures is performed with adequate denture cleansing in order to prevent associated oral and systemic diseases^6^.

Although most patients clean their dentures by manual brushing, this method when used isolated has been considered one of the least efficient for biofilm control^12^. The brushing method requires manual dexterity and visual acuity which are usually compromised in elderly individuals^12^. Furthermore, the microorganisms embedded in the biofilm become partially protected by the shear forces of the toothbrush^2^. The irregularities and porosities of acrylic bases also favor the penetration of microorganisms, which raise difficulties in cleaning them exclusively by brushing, and therefore the prostheses can become a source of infection and re-infection of supporting tissues^12^. Consequently, for biofilm control, daily immersion in cleaning solutions has been suggested to complement denture hygiene and this combination has demonstrated greater results when compared to brushing alone^10^.

Various denture cleansers are commercially available with different active agents, including hypochlorite, peroxides, enzymes and acids. Homemade solutions are often adopted by patients who perform a daily chemical method of denture cleaning because of their easy of acquisition and low cost. Sodium hypochlorite is a chemical solution that is routinely recommended for cleaning dentures and has the ability to dissolve mucin and other organic components being highly effective at removing light stains. Also, sodium hypochlorite solution has bactericidal and fungicide action^6^. However, this chemical agent corrodes metal components of prostheses and degrades the acrylic resin components, causing color changes (lightening) and an increase in surface roughness^6^ ^,^ ^11^. Chlorhexidine also been suggested as an adjunct to denture cleansing by brushing. This chemical agent is widely used both for prevention and treatment of oral infections, as antiseptics and disinfectants for removable dentures^3^ ^,^ ^7^. Chlorhexidine gluconate presents wide spectrum of action and significant substantivity, hence it can be used effectively when mixed bacterial and fungal bioﬁlms are present^8^. Alkaline peroxides are also commonly used as denture cleansers and produce an effervescent alkaline solution of hydrogen peroxide, containing active oxygen, when in contact with water. The effervescence presents a mechanical action for removing debris, and the oxygen has antimicrobial and stain-removing effects. Some products also contain enzymes to failure biofilm proteins^10^ ^,^ ^14^. Seeking an alternative to commonly known denture cleansers, new materials with antimicrobial action have been investigated such as the oil derived from castor bean (*Ricinus communis*) evaluated by Salles, et al.^18^ (2015) in this issue. *R. communis* oil solution presents biocompatibility and bactericidal and fungicidal effects. It is colorless and does not have an unpleasant smell. These characteristics along with detergent action make its use in denture cleaning possible^9^.

As control of biofilm is a constant concern, *in vivo *and *in vitro* studies^10^ ^,^ ^16^ ^,^ ^18^ ^,^ ^19^ have been continuously developed to evaluate the efficiency of cleaning agents for dentures. Pellizzaro, et al.^16^ (2012) investigated *in vitro* the effectiveness of combining brushing and cleansing agents (dentifrice, 2% chlorhexidine gluconate, 1% sodium hypochlorite and abrasive-free foaming antibacterial denture cleanser - Polident fresh cleanse^®^) in killing *C. albicans* biofilm on acrylic resin disks. They concluded that the use of the combined method of brushing with cleansing agents is an effective method to reduce *C. albicans* biofilm, being 2% chlorhexidine gluconate and 1% sodium hypochlorite the most effective solutions. The aim of the study by de Sousa Porta, et al.^19^ (2015) was to evaluate, *in vivo*, the efficacy of 0.5% sodium hypochlorite for 3 min over 90 days as a denture cleanser and its effect on color stability and surface roughness of complete dentures. Patient satisfaction with the denture cleaning method was also assessed. The authors found that 0.5% sodium hypochlorite solution in conjunction with brushing was effective in reducing microorganism numbers without causing significant color or roughness changes. Moreover, participants reported high levels of satisfaction with the cleaning results. Lucena-Ferreira, et al.^10^ (2014) investigated *in vitro* the effect of daily exposure for 3 min in an alkaline peroxide enzyme-containing commercial denture cleanser (Polident^®^ 3 Minute) on a multispecies biofilm (five bacteria and *Candida albicans*) formed on cylindrical specimens of acrylic resin. The daily exposure of multispecies biofilms to denture cleanser reduced the number of total microorganisms but increased *C. albicans* counts. Thus, the authors suggested that daily use of denture cleanser is an effective method for controlling bacteria in biofilm, but it can potentially select *C. albicans*, an important etiological agent of oral candidosis. In this issue, Salles, et al.^18^ (2015), by a crossover randomized clinical trial, aimed to evaluate the antimicrobial activity of sodium hypochlorite (0.25% and 0.5%) and *R. communis* oil (10%) solutions along with the mechanical method of brushing against *Streptococcus mutans*, *Candida *spp., and Gram-negative microorganisms. All three solutions showed antimicrobial activity against *S. mutans*. For *Candida *spp., *R. communis* oil and 0.25% sodium hypochlorite solutions showed similar effect while 0.5% sodium hypochlorite showed superior activity. Both sodium hypochlorite solutions showed antimicrobial action against gram-negative microorganisms. The *Candida *species most frequently isolated was *C. albicans*, followed by *C. tropicalis* and *C. glabrata*. The authors concluded 0.5% sodium hypochlorite solution was the most effective and might be used for short immersions along with brushing to control denture biofilm formation. As *R. communis *demonstrated antimicrobial activity against *S. mutans* and *Candida* spp., this solution also was recommended as an auxiliary method of denture cleaning along with brushing^18^.

Adoption of routine oral hygiene practices including mechanical cleaning in conjunction with immersion in denture cleansers is essential to ensure the careful daily removal of oral and denture biofilms. This is the key to minimize the risk of opportunistic infections, to contribute to good oral and overall systemic health and to maintain an aesthetically pleasing, odor-free appliance. More than being able to recommend a certain denture cleanser, the clinician should advise patients on the importance of removing the denture biofilm for the maintenance of oral and general health as well instruct the denture wearers to use the denture cleanser it correctly as co-adjuvant method for denture cleaning.
